# Cystic artery pseudoaneurysm secondary to acute cholecystitis: an unusual cause for haemobilia

**DOI:** 10.1259/bjrcr.20150423

**Published:** 2016-05-24

**Authors:** Thomas Charles Hall, Walter Sprenger De Rover, Said Habib, Maruti Kumaran

**Affiliations:** Department of Radiology, Queen’s Medical Centre, Nottingham, UK

## Abstract

Pseudoaneurysm of the cystic artery is a rarely described cause of haemobilia. We report the unusual presentation of upper gastrointestinal haemorrhage due to a pseudoaneurysm of the cystic artery secondary to acute cholecystitis that was complicated by gallbladder perforation and liver abscess in an 88-year-old male. The original CT scan had demonstrated a high density focus in the gallbladder neck that was thought to represent a calculus. Selective embolization of the cystic artery resulted in cessation of the haemorrhage. Owing to the patient’s frailty and comorbidities, he was not considered suitable for cholecystectomy. The case emphasizes the need for a high level of awareness of pseudoaneurysmal disease in association with inflammatory conditions.

## Summary

Pseudoaneurysms of the cystic artery are uncommon, having been reported in only 16 cases in the English literature.^1,2^ Visceral pseudoaneurysmal disease is usually seen in the context of a provoking inflammatory condition such as acute pancreatitis or due to iatrogenic surgical insult.^3,4^

Spontaneous intracholecystic haemorrhage is uncommon and is usually described in patients with blood dyscrasia, vascular disease, hepatobiliary tumours and parasitic infestation.^5,6^ We describe a very unusual case of a haemorrhaging pseudoaneurysm of the cystic artery secondary to acute cholecystitis causing upper gastrointestinal haemorrhage. In the writing of this report, the principles of the Declaration of Helsinki were followed.

## Case report

An 88-year-old male presented to the emergency department complaining of upper abdominal pain, malaise, fever (38. 1 °C) and rigors of increasing severity for the preceding 3 weeks. There was no prior history of abdominal surgery or trauma. He was febrile on presentation, with diffuse tenderness and guarding in his upper abdomen. His biochemistry revealed normal liver function except for neutrophil leukocytosis (8.2 K µl^–1^) and thrombocythaemia (487 × 10^9 ^l^–l^). His coagulation tests were within normal limits.

**Figure 1.**
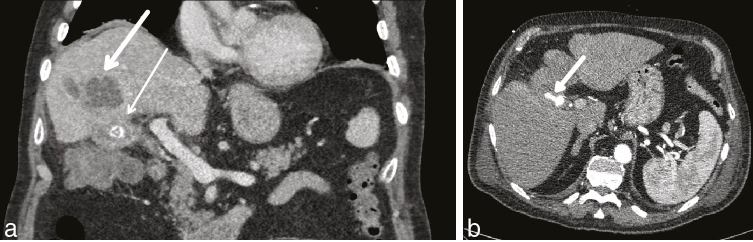
Portal venous phase CT scan. (a) Thick white arrow points to liver abscess; thin white arrow points to high density within the gallbladder. (b) Arterial phase 1 week later (arrow denotes the pseudoaneurysm).

A CT scan demonstrated a thick-walled gallbladder containing numerous radiopaque calculi that was consistent with cholecystitis. This was complicated by an adjacent liver abscess and the suggestion of a localized gallbladder perforation. An ultrasound-guided drain insertion into the liver abscess was attempted and pus was aspirated; however, the patient did not tolerate the procedure and it was abandoned. There was no instrumentation of the gallbladder during this procedure. Initial plans to also drain the gallbladder were abandoned owing to patient refusal. However, his condition deteriorated with worsening sepsis and a repeat CT scan demonstrated increasing dimensions of the liver abscess and a high density focus within the gallbladder neck, at this point thought to represent a calculus (Figure 1a). Repeat ultrasound-guided drainage was attempted under sedation, which was successful, and blood-stained pus was aspirated. The blood staining was thought to be traumatic in nature.

**Figure 2.**
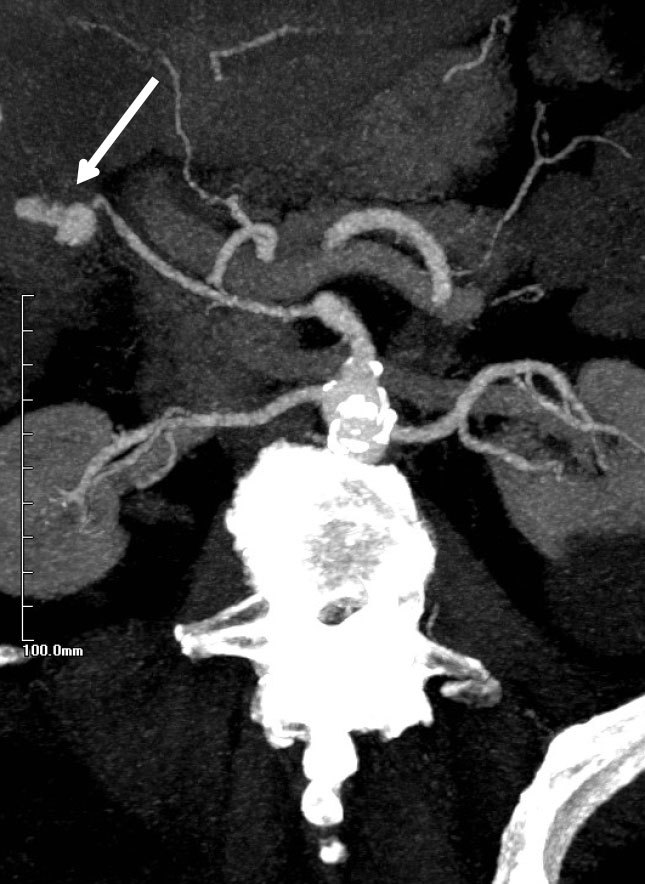
Arterial phase maximum intensity projection of the pseudoaneurysm (denoted by the white arrow).

The patient then developed melena and anaemia, and had an unremarkable upper gastrointestinal endoscopy. A CT angiogram demonstrated a 26 × 17 mm pseudoaneurysm arising from the cystic artery and haemobilia (Figure 1b). In retrospect, this was seen on prior imaging, where the lesion was misinterpreted as a calculus in the gallbladder neck. This CT scan revealed that the high density focus (pseudoaneurysm) had increased in size following the previous CT scan taken 1 week prior. Arterial anatomy showed a cystic artery that originated from the right hepatic artery as a branch of the superior mesenteric artery (Figure 2).

**Figure 3.**
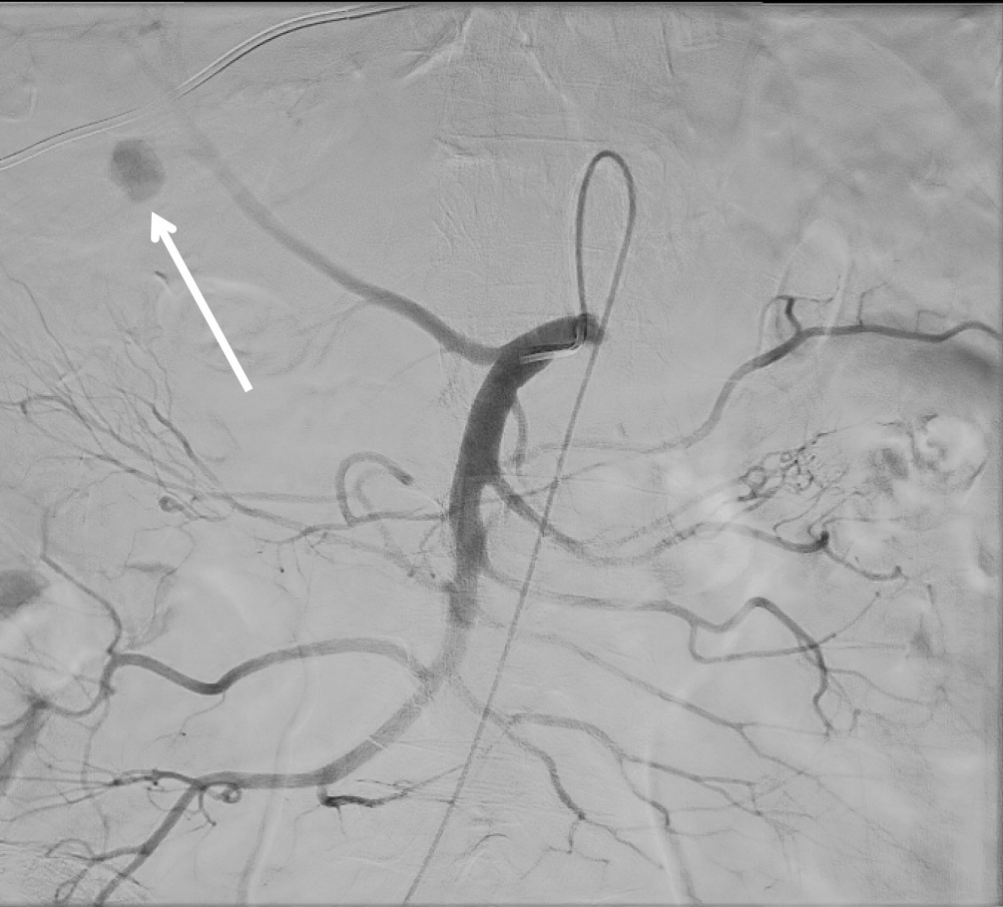
Selective catheterization of the cystic artery. Arrow indicates the pseudoaneurysm.

In our institution, we would usually manage such pseudoaneurysms by either selectively coiling the feeding artery, filling the pseudoaneurysm sac with coils, placing a covered stent across the feeder or using embolic agents or thrombin. In this case, the aim was to selectively coil the feeding vessel via right common femoral artery puncture; however, it was not technically possible to engage a catheter into the sac and during the process of negotiation, the sac ruptured. The pseudoaneurysm was then embolized by instilling a mixture of 2 : 4 cyanoacrylate and  lipiodol (Figure 3) into the cystic artery as an instant embolic agent and accept the risk of gallbladder necrosis. The right hepatic artery was preserved.

The patient made an uneventful immediate recovery following the embolization and appropriate antimicrobial treatment. The liver drain was removed a week later and he was discharged with oral antibiotics. Owing to his comorbidities and frailty, it was decided not to proceed to cholecystectomy. A follow-up ultrasound showed regression of the liver abscess and collapse of the gallbladder containing calculi.

## Discussion

Despite the high prevalence of hepatobiliary infection, there have been no prior reported cases of cystic artery pseudoaneurysm causing haemobilia in our institution. Haemobilia usually presents as upper abdominal pain, obstructive jaundice and gastrointestinal haemorrhage, known as Quincke’s triad.^7^ Although the case we present was not jaundiced, this is not an uncommon finding. A recent case series of 16 patients by Akatsu and colleagues^1^ reported that 38% of patients with cystic artery pseudoaneurysm were not jaundiced. In addition, they reported that in 56% of patients, the pseudoaneurysm was located inside the gallbladder and ranged in size from 0.2 to 4 cm (median 2.5 cm). The majority of patients included in their study had undergone cholecystectomy with or without temporary biliary drainage.^1^ In the present case, embolization and drainage of the liver abscess alone was performed owing to the patient’s frailty and comorbidity. Prior case reports have also described the successful management of cystic artery pseudoaneurysm with selective cystic artery embolization.^1^

It seems likely that the origin of the pseudoaneurysm is secondary to erosion of the cystic artery wall owing to the inflammatory process associated with cholecystitis. The friability of the pseudoaneurysm and its subsequent rupture during embolization is supportive of its mycotic aetiology. Given that cholecystitis is a common presentation, it may seem strange that cystic artery pseudoaneurysm is so uncommon. It has been postulated by other authors that the inflammatory reaction predisposes to early thrombosis of the pseudoaneurysm, preventing its haemorrhagic rupture and associated sequalae.^8^

## Learning points

1. The appreciation of the rare occurrence of a cystic artery pseudoaneurysm in the context of calculus and acute cholecystitis is important to avoid the assumption that the high density seen within the gallbladder on a contrast-enhanced CT scan is due to calculi or biliary sludge.
2. Selective angiography followed by embolization of the affected artery resulted in control of the haemorrhage.

## Consent

Written informed consent was obtained from the patient for publication of this case report, including accompanying images.

## References

[1] Akatsu T, Tanabe M, Shimizu T, Handa K, Kawachi S, Aiura K, et al Pseudoaneurysm of the cystic artery secondary to cholecystitis as a cause of hemobilia: report of a case. Surg Today 2007; 37: 412–17. 17468824 10.1007/s00595-006-3423-2

[2] Muñoz-Villafranca C, García-Kamirruaga Í, Góme-García P, Atín-del-Campo V, Bárcena-Robredo V, Aguinaga-Alesanco A, et al Pseudoaneurysm of the cystic artery: an uncommon cause of upper gastrointestinal bleeding in a case of xanthogranulomatous cholecystitis. Rev Esp Enferm Dig 2015; 107: 375–6. 26031867

[3] Curet P, Baumer R, Roche A, Grellet J, Mercadier M Hepatic hemobilia of traumatic or iatrogenic origin: recent advances in diagnosis and therapy, review of the literature from 1976 to 1981. World J Surg 1984; 8: 2–8. 6367231 10.1007/BF01658356

[4] Croce MA, Fabian TC, Spiers JP, Kudsk KA Traumatic hepatic artery pseudoaneurysm with hemobilia. AM J Surg 1994; 168: 235–8. 8080059 10.1016/s0002-9610(05)80193-x

[5] Mori M, Kohzaki S, Makino K, Amamoto Y, Mori M, Kanbara C, et al Spontaneous intracholecystic hemorrhage due to polyarteritis nodosa. J Comput Assist Tomogr 1998; 22: 730–1. 9754107 10.1097/00004728-199809000-00012

[6] Laing FC, Frates MC, Feldstein VA, Goldstein RB, Mondro S Hemobilia: sonographic appearances in the gallbladder and biliary tree with emphasis on intracholecystic blood. J Ultrasound Med 1997; 16: 537–43. 9315209 10.7863/jum.1997.16.8.537

[7] Nakajima M, Hoshino H, Hayashi E, Nagano K, Nishimura D, Katada N, et al Pseudoaneurysm of the cystic artery associated with upper gastrointestinal bleeding. J Gastroenterol 1996; 31: 750–4. 8887048 10.1007/BF02347630

[8] Barba CA, Bret PM, Hinchey EJ Pseudoaneurysm of the cystic artery: a rare cause of hemobilia. Can J Surg 1994; 37: 64–6. 8306223

